# Lung Scintigraphy for Pulmonary Embolism Diagnosis in COVID-19 Patients: A Multicenter Study

**DOI:** 10.2967/jnumed.121.262955

**Published:** 2022-07

**Authors:** Pierre-Yves Le Roux, Pierre-Benoit Bonnefoy, Achraf Bahloul, Benoit Denizot, Bertrand Barres, Caroline Moreau-Triby, Astrid Girma, Amandine Pallardy, Quentin Ceyrat, Laure Sarda-Mantel, Micheline Razzouk-Cadet, Reka Zsigmond, Cachin Florent, Gilles Karcher, Pierre-Yves Salaun

**Affiliations:** 1 Service de Médecine Nucléaire, Université Européenne de Bretagne, Université de Brest, EA3878 (GETBO) IFR 148, CHRU de Brest, Brest, France;; 2 Service de Médecine Nucléaire, CHU de Saint-Etienne, Saint-Etienne, France;; 3 Service de Médecine Nucléaire, CHRU Nancy, Nancy, France;; 4 Service de Médecine Nucléaire, Centre Hospitalier Alpes Léman, Contamine sur Arve, France;; 5 Service de Médecine Nucléaire, Centre Jean Perrin, Clermont Ferrand, France;; 6 Service de Médecine Nucléaire, Groupe Hospitalier de l’Est, Bron, France;; 7 Service de Médecine Nucléaire, Hôpital Foch, Suresne, France;; 8 Service de Médecine Nucléaire, CHU de Nantes, Nantes, France;; 9 Centre d’Imagerie Fonctionnelle, Bordeaux, France;; 10 Service de Médecine Nucléaire, Hôpital Lariboisière, APHP, Paris, France;; 11 Service de Medecine Nucléaire, CHU Nice, Nice, France; and; 12 Centre d’Imagerie Nucléaire, Annecy, France

**Keywords:** pulmonary embolism, ventilation perfusion scintigraphy, SPECT, COVID-19

## Abstract

In patients with novel coronavirus disease 2019 (COVID-19) referred for lung scintigraphy because of suspected pulmonary embolism (PE), there has been an ongoing debate within the nuclear medicine community as to whether and when the ventilation imaging should be performed. Indeed, whereas PE diagnosis typically relies on the recognition of ventilation–perfusion (V/P) mismatched defects, the ventilation procedure potentially increases the risk of contamination to health-care workers. The primary aim of this study was to assess the role of ventilation imaging when lung scintigraphy is performed because of suspected PE in COVID-19 patients. The secondary aim was to describe practices and imaging findings in this specific population. **Methods:** A national registry was created in collaboration with the French Society of Nuclear Medicine to collect lung scans performed on COVID-19 patients for suspected PE. The practices of departments were assessed regarding imaging protocols and aerosol precautions. A retrospective review of V/P SPECT/CT scans was then conducted. Two physicians masked to clinical information reviewed each case by sequentially viewing perfusion SPECT, perfusion SPECT/CT, and V/P SPECT/CT images. The scans were classified into 1 of the 4 following categories: patients for whom PE could reasonably be excluded on the basis of perfusion SPECT only, perfusion SPECT/CT, or V/P SPECT/CT and patients with mismatched defects suggestive of PE according to the European Association of Nuclear Medicine criteria. **Results:** Data from 12 French nuclear medicine departments were collected. Lung scans were performed between March 2020 and April 2021. Personal protective equipment and dedicated cleaning procedures were used in all departments. Of the 145 V/Q SPECT/CT scans included in the central review, PE could be excluded using only perfusion SPECT, perfusion SPECT/CT, or V/P SPECT/CT in 27 (19%), 55 (38%), and 45 (31%) patients, respectively. V/P SPECT/CT was positive for PE in 18 (12%) patients, including 12 (67%) with a low burden of PE (≤10%). **Conclusion:** In this population of COVID-19 patients assessed with lung scintigraphy, PE could confidently be excluded without the ventilation imaging in only 57% of patients. Ventilation imaging was required to confidently rule out PE in 31% of patients. Overall, the prevalence of PE was low (12%).

## Visual Abstract

**Figure:**
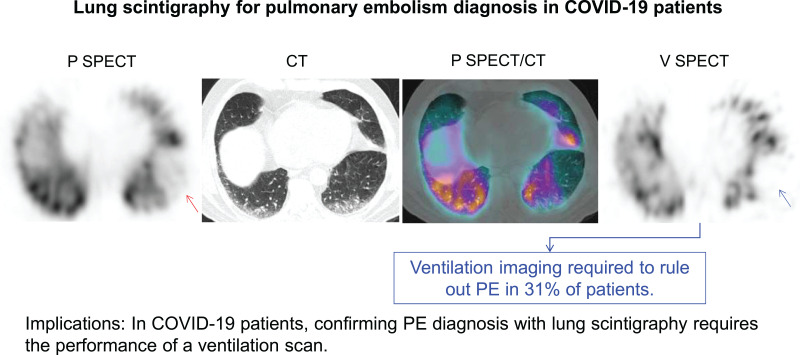

A frequent complication of novel coronavirus disease 2019 (COVID-19) is coagulopathy, which manifests in the form of both microthrombosis and venous thromboembolism (*1*). Lung ventilation–perfusion (V/P) scintigraphy is a well-established test for diagnosis of pulmonary embolism (PE) (*2*). Diagnostic strategies based on lung scintigraphy have been widely validated in large studies on diagnostic accuracy (*3*) and management outcome (*4*–*7*), in which interpretation of the lung scan was based on recognition of wedge-shaped perfusion mismatched defects, that is, perfusion defects with normal ventilation.

However, the ventilation procedure increases the potential risk of contamination by the aerosol secretion and the expired air to health-care workers and to other patients (*8**,**9*). As a result, a variety of strategies has been proposed in the nuclear medicine literature regarding performing lung scintigraphy on COVID-19 patients with suspected acute PE (*10*). Some have proposed omitting the ventilation scan and performing only perfusion scinigraphy or perfusion SPECT/CT (*11*–*14*), arguing that this approach allows sufficient diagnostic performance while reducing the risk of contamination. Others have recommended systematically maintaining the standard V/P procedure, with appropriate aerosol precautions for health-care workers (*15*–*17*). The rational for this approach is that a wide proportion of patients with confirmed COVID-19 infection and symptoms suggestive of acute PE will have abnormal findings on the perfusion scan and that not performing a ventilation scan is associated with an unacceptably high risk of false-positive results. Finally, some have proposed an intermediate approach with a standalone perfusion planar or SPECT/CT scan, followed, only when necessary, by a ventilation scan (*18**,**19*). However, although various conflicting opinions and recommendations have been published on performing lung scintigraphy on COVID-19 patients, there are currently no factual data on this specific population to support recommendations to the nuclear medicine community.

The primary aim of this study was to assess the role of ventilation imaging when performing lung scintigraphy for suspected PE in COVID-19 patients. The secondary aim was to describe practices and imaging findings in this specific population.

## MATERIALS AND METHODS

### Collection of Data

A national registry was created at the initiative of the French Society of Nuclear Medicine working group on lung scintigraphy to collect lung scans performed on COVID-19 patients for suspected PE. In collaboration with the French Society of Nuclear Medicine, the information was circulated to its memberships via 3 sets of e-mails in April, June, and December 2020. Physicians who had notified the investigators that they had cases of COVID-19 were then contacted to participate in the study. The protocol was approved by the nuclear medicine research ethics committee (CEMEN [Comité d’Ethique pour la Medecine Nucléaire] 2021-01). Informed consent was obtained from all participants.

### Evaluation of Practices

The general practices of nuclear medicine facilities were assessed using a standardized questionnaire. The questionnaire encompassed aerosol precautions for the health-care workers (type of mask, goggles, gloves, long cap, gown), cleaning procedures, organizational adaptations in the nuclear medicine facility, and imaging protocols for the performance of lung scintigraphy on patients with confirmed COVID-19 and suspected acute PE. Nuclear medicine facilities were also questioned about any possible contamination of a health-care worker after managing a patient with COVID-19 disease.

Individual data from patients with confirmed COVID-19 who underwent lung scintigraphy for suspected acute PE were then collected. These patients include those who underwent various acquisition protocols, including planar scintigraphy (with or without ventilation), perfusion SPECT/CT (without ventilation), or V/P SPECT/CT scans. Only patients still considered contagious were analyzed, that is, patients for whom the lung scan was typically performed within 14 d after initiation of symptoms. For each patient, demographic information and lung scan images were collected.

### V/P SPECT/CT Central Review

To assess the role of ventilation imaging in COVID-19 patients with suspected PE, a centralized retrospective review of scans with a complete V/P SPECT/CT protocol was then conducted. Scans of patients who underwent a planar or a perfusion SPECT/CT protocol (without ventilation) were not included in this retrospective review. A consensus reading of images was performed by 2 board-certified nuclear medicine physicians masked to clinical information. Each case was reviewed by sequentially using perfusion SPECT, perfusion SPECT/CT, and V/P SPECT/CT images. First, only perfusion SPECT images were used. Scans with normal perfusion were classified as negative for PE. Second, in patients with an abnormal result on perfusion SPECT, CT images were added. Scans whose perfusion defects were matched with CT findings were classified as negative for PE. Finally, in patients with mismatched perfusion SPECT/CT defects, ventilation SPECT images were added for interpretation. V/P SPECT/CT scans were then interpreted as negative or positive for PE according to the presence of V/P mismatched defects suggestive of acute PE according to the European Association of Nuclear Medicine criteria (*20**,**21*). Accordingly, scans were classified into 1 of the 4 following categories: patients for whom PE could reasonably be excluded on the basis of perfusion SPECT only, perfusion SPECT/CT, or V/P SPECT/CT and patients with mismatched V/P defects suggestive of PE. In patients with a positive V/Q SPECT/CT result, the number of segmental and subsegmental mismatched defects was recorded.

## RESULTS

Data from 183 patients with confirmed COVID-19 disease who underwent lung scintigraphy for suspected acute PE between March 2020 and April 2021 in 12 French nuclear medicine departments were collected.

### Evaluation of Practices

The practices of the 12 nuclear medicine departments are presented in Table 1. Personal protective equipment and dedicated cleaning and disinfecting procedures were used in all departments. Scans were formed in a dedicated room in 8 (67%) centers and at the end of the day in 10 (83%). Eleven (92%) centers systematically performed a ventilation scan before the perfusion acquisition. V/P SPECT/CT was the first-line imaging procedure in 8 (75%) centers. Of the 12 nuclear medicine facilities, there was no reported case of possible contamination of a health-care worker after managing a COVID-19 patient.

**TABLE 1.** Practices of Nuclear Medicine Facilities

Practice	Centers (*n*)
Aerosol precautions	12 (100%)
Mask	12 (100%)
Filtering facepiece 1	1 (8%)
Filtering facepiece 2	11 (92%)
Goggles	12 (100%)
Long cap	12 (100%)
Gloves	12 (100%)
Gown	12 (100%)
Specific COVID-19 cleaning and disinfecting procedures	12 (100%)
Organizational adaptation in nuclear medicine facility	
Camera or room dedicated to COVID-19 patients	8 (67%)
Scans performed at end of day	10 (83%)
Dedicated circuit within nuclear medicine department	3 (25%)
Standard lung scan protocol for COVID-19 patients	
Systematic ventilation scan	11 (92%)
Technegas	8 (73%)
^81m^Kr gas	3 (27%)
First-line imaging	
Planar	3 (25%)
SPECT	0 (0%)
SPECT/CT	9 (75%)

Of the 183 patients, 117 (64%) were female. The median age was 74 y (range, 15–102 y). Sixty-eight (37%) were older than 80 y, and 26 (14%) were older than 90 y. A ventilation scan was performed on 168 patients (92%), using Technegas (Cyclomedica Australia Pty. Ltd.) aerosol in 144 (86%) and ^81m^Kr gas in 24 (14%). Twenty-four (13%) had only a planar scan, and 159 (87%) had a SPECT/CT scan.

### V/Q SPECT/CT Interpretation

**FIGURE 1.**
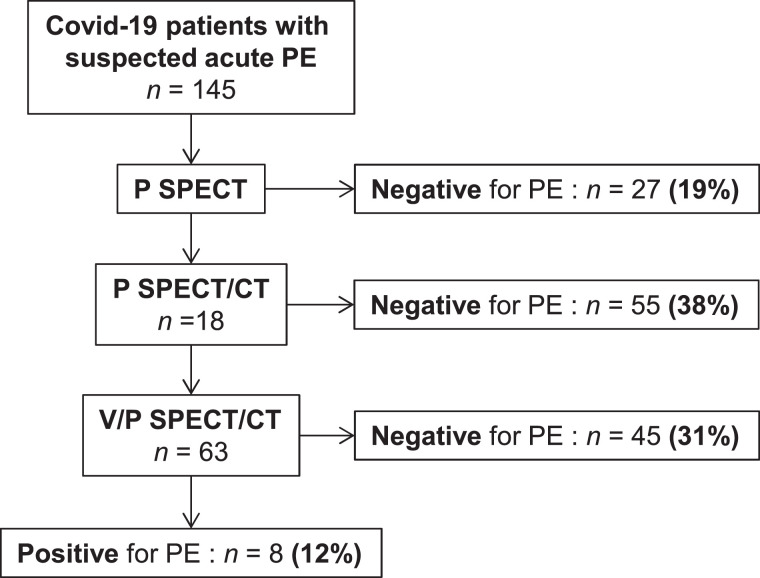
Results of central review. P = perfusion.

**FIGURE 2.**
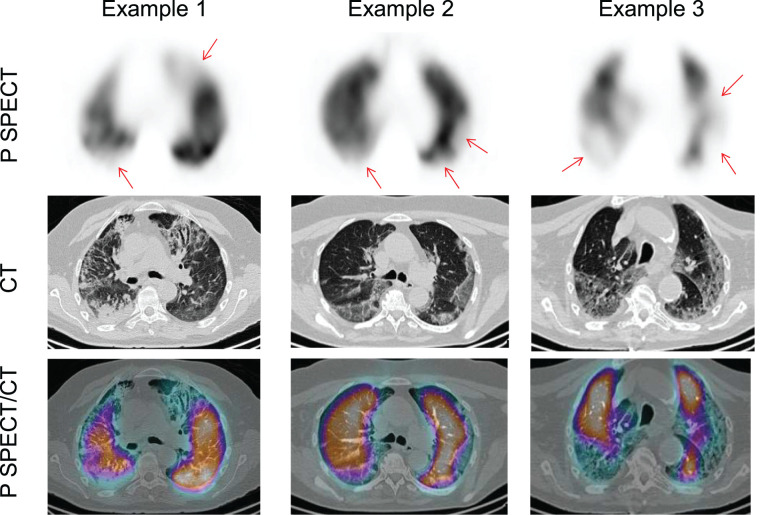
Examples of negative perfusion SPECT/CT results, with perfusion defects (arrows) matched with chest CT findings of COVID-19 disease. P = perfusion.

**FIGURE 3.**
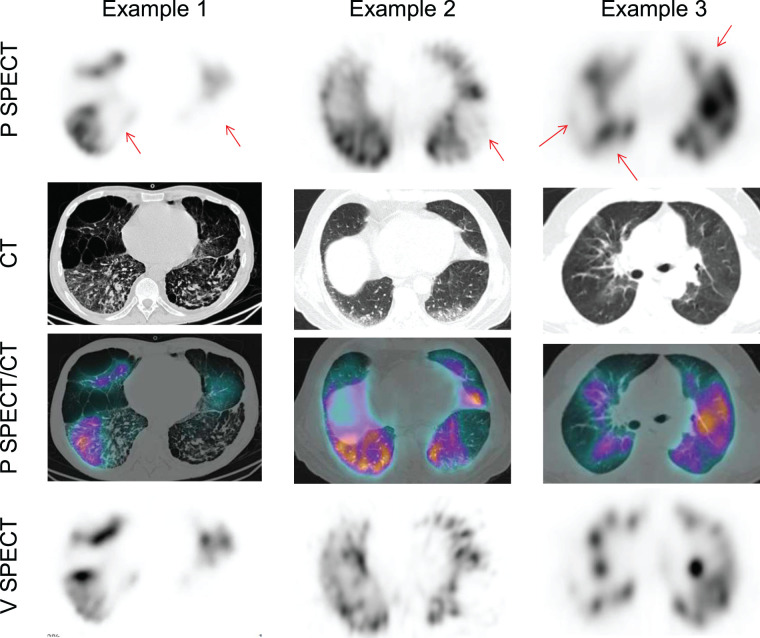
Examples of false-positive perfusion SPECT/CT results. Perfusion SPECT images showed perfusion defects (arrows), without significant abnormality on CT images. Perfusion SPECT/CT scans would therefore have been read as positive for PE. However, ventilation SPECT demonstrated matched defects. V/Q SPECT/CT scans were therefore interpreted as negative for PE. P = perfusion; V = ventilation.

**FIGURE 4.**
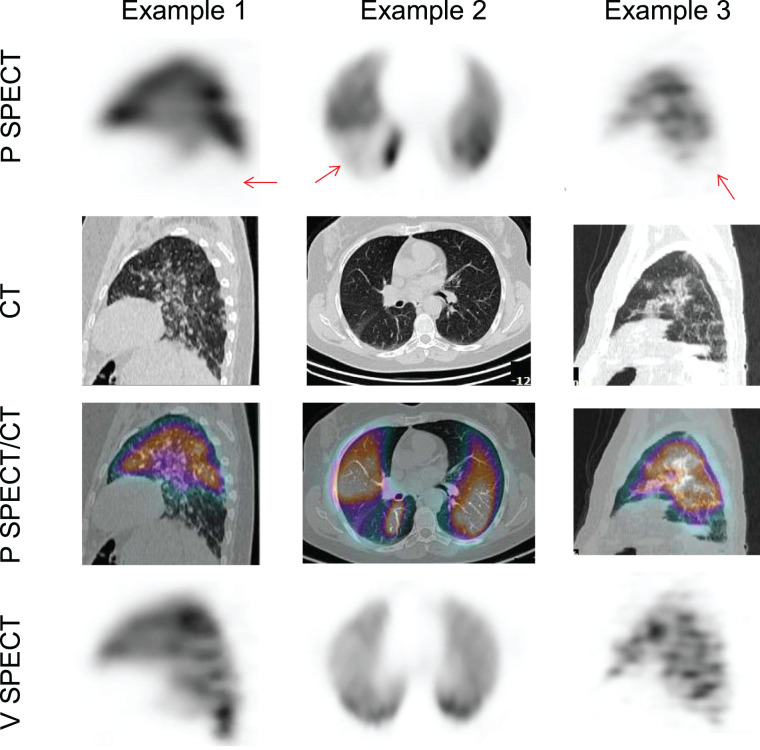
Examples of positive V/P SPECT/CT results. Perfusion SPECT images showed perfusion defects (arrows), whereas coregistered ventilation SPECT images showed normal ventilation (mismatched V/P defects). P = perfusion; V = ventilation.

In total, 145 complete V/P SPECT/CT scans were centrally reviewed. Of them, PE could be excluded using only perfusion SPECT, perfusion SPECT/CT, or V/P SPECT/CT in 27 (19%), 55 (38%), and 45 (31%) patients, respectively (Fig. 1). Examples of negative perfusion SPECT/CT findings for which perfusion defects matched chest CT findings of COVID-19 disease are shown in Figure 2. Examples of false-positive results using the perfusion SPECT/CT approach are presented in Figure 3. V/P SPECT/CT imaging was positive for PE in 18 (12%) patients. The burden of PE was 10% or less in 12 (67%) patients, more than 10% but no more than 20% in 4 (22%) patients, and more than 20% in 2 (11%) patients. Examples of positive V/P SPECT/CT scans are shown in Figure 4.

## DISCUSSION

In this population of COVID-19 patients assessed with lung scintigraphy, PE could confidently be excluded without ventilation in 57% of patients. In contrast, ventilation imaging was required in the remaining 43% to confidently rule out (31%) or confirm (12%) the PE diagnosis.

Interpretation of lung scans for PE typically relies on recognition of V/P mismatched defects (*22*). The sensitivity of the test—that is, the ability to detect PE—relies on recognition of defects on perfusion images. Normal findings on a perfusion scan have been shown to safely rule out a PE diagnosis (*4*–*7*). However, there are many nonthromboembolic lung diseases that may cause perfusion defects. To increase the specificity—that is, the ability of the test to detect whether a patient is free of disease—perfusion scintigraphy has to be compared with ventilation images to differentiate V/P mismatched defects highly suggestive of acute PE from V/P matched defects of other etiologies. With the advent of SPECT/CT cameras has come a proposal to coregister SPECT data with a low-dose CT scan to further increase the specificity of the test. Some groups have proposed replacing the ventilation SPECT.

In our series, only 19% of COVID-19 patients had strictly normal results on the perfusion SPECT scan. In this specific population of COVID-19 patients with suspected acute PE, there is therefore a high likelihood (nearly 4 in 5 chance) that an approach consisting of performing only a perfusion lung scan, without CT images, will be nondiagnostic. When perfusion SPECT images were coregistered with a low-dose CT scan, 38% more scans became diagnostic, as all perfusion defects could confidently be explained by non-PE morphologic findings. Overall, perfusion SPECT/CT imaging would have allowed confident exclusion of PE in 57% of COVID-19 patients, without exposing the health-care workers to an increased risk of contamination due to the ventilation procedure. Nevertheless, the benefit of omitting the ventilation procedure should be balanced against the risk of increasing the number of patient transfers through health-care facilities and increasing the amount of contact between individuals.

In the remaining 43% of patients, a ventilation scan was required to exclude (31%) or confirm (12%) the diagnosis of PE. Most patients with a positive scan result did not have a massive PE with multiple wedge-shaped perfusion defects highly suggestive of PE irrespective of the ventilation scan. On the contrary, the ventilation study was helpful in most positive scans, which demonstrated only 1 or 2 segmental perfusion mismatched defects.

Our data confirm the high risk of a false-positive result when omitting the ventilation scan. In a retrospective series of 393 patients assessed by V/P SPECT imaging for suspected PE, 15% of patients with a negative V/P SPECT result would have been wrongly diagnosed with PE using a perfusion SPECT/CT approach (*23*). In another series of 81 patients, the specificity decreased from 100% with V/P SPECT/CT to 51% with perfusion SPECT/CT (*24*). Similarly, in a study of 93 patients, 17% of V/Q SPECT scans with negative results were falsely positive when compared with perfusion SPECT/CT (*25*). The risk of false-positive results was even higher in our population of COVID-19 patients, probably because most patients had COVID-19 parenchymal lung disease and thus abnormal lung perfusion. Also noteworthy is the age of the population, with a median of 74 y, and with 37% of patients older than 80 y, increasing the likelihood of abnormal perfusion from any lung disease.

A false-positive diagnostic test may have major consequences for patients with suspected acute PE. Indeed, current clinical guidelines suggest extended anticoagulation in patients with no identifiable risk factor or with a minor transient or reversible risk factor for the index PE event (*2*). Accordingly, a false-positive lung scintigraphy result will mean lifelong anticoagulant therapy and its risks of bleeding for many patients (*15*).

Surprisingly, the prevalence of positive scans was low (12%) in this population of COVID-19 patients with suspected PE assessed with V/Q SPECT/CT imaging. This prevalence is much lower than that of PE across studies on V/Q SPECT in non–COVID-19 patients—a prevalence that ranges from 17% to 54% (median, 26%) (*26*). This prevalence is also low as compared with studies assessing other imaging tests for PE (e.g., CT pulmonary angiography or planar V/P scanning). In a metaanalysis performed before the COVID-19 pandemic including 29,684 patients from 49 studies, the pooled prevalence of PE was 22.6% (*27*). As it is now well established that COVID-19 predisposes patients to thrombotic events (*28*), this prevalence probably means that there is more suspected PE in COVID-19 patients than in non–COVID-19 patients, likely resulting from the combination of poorly explained respiratory symptoms in patients at risk for venous thromboembolism. Furthermore, in patients positive for PE, the burden of disease was low, with only 11% of patients having a PE burden of more than 20%. It is likely that most patients admitted to intensive care units or with signs of hemodynamic instability were referred for CT pulmonary angiography rather than for lung scintigraphy (*29*).

An assessment of general practices showed a major trend among the 12 French institutions. The typical examination was a V/P SPECT/CT acquisition as commonly performed on non–COVID-19 patients (*30*), with personal protective equipment for the health-care workers and dedicated cleaning procedures. Most institutions obtained acquisitions on a dedicated camera at the end of the day. No case of possible contamination of a health-care worker after managing a COVID-19 patient was reported, although this finding should be interpreted with caution.

Our study had some limitations. First, the results of the central review were not compared with an independent reference standard, and patient follow-up data were not collected. Accordingly, we cannot assert that all patients with a negative scan result did not have PE and that all patients with a positive scan result did have PE. Despite these limitations resulting from the retrospective design of this multicenter study based on a national registry, this was, to our knowledge, the first large series that assessed the usefulness of the ventilation scan in COVID-19 patients. Major trends have emerged with regard to the prevalence of PE and the proportion of inconclusive results with the various lung scan protocols. These results may assist nuclear physicians in the performance of lung scintigraphy on COVID-19 patients with suspected acute PE, according to their own local situation. Second, assessment of general practices probably does not reflect the reality in nuclear medicine facilities around the world. Indeed, the French Society of Nuclear Medicine working group on lung scintigraphy recommended that ventilation scans be performed on COVID-19 patients—a recommendation that is likely to have influenced practices in France. Furthermore, institutions that followed these recommendations may have been more inclined to participate in the study. However, our results show that the usual V/P SPECT/CT approach can be used in daily practice on COVID-19 patients. In our series, there was no reported case of possible contamination of a health-care worker after managing a COVID-19 patient. However, this finding was just observational, and we did not perform formal testing such as swabbing for virus. Accordingly, we cannot draw any conclusions on the risk of contamination. Third, according to the European Association of Nuclear Medicine guidelines for lung scintigraphy (*20*) or the French Society of Nuclear Medicine guidelines for lung scintigraphy protocols (*31*), the CT was performed as a low-dose scan during continuous shallow breathing. Accordingly, the CT scans do not fulfill the criteria for diagnostic quality. Optimization of acquisition and reconstruction parameters could enhance the diagnostic performance of the CT scans. Finally, we performed a consensual interpretation of scans and did not assess interobserver agreement.

## CONCLUSION

In 57% of COVID-19 patients assessed with lung scintigraphy, PE could be confidently excluded without a ventilation study. This approach allows us to limit the risk of contamination to health-care workers but should be balanced against the risk of increasing transfers of the remaining 43% of patients through health-care facilities. Indeed, our study clearly confirmed the high risk of false-positive results when omitting the ventilation study, a risk that appears unacceptable given the risk of bleeding and the trend toward an indefinite duration of anticoagulation in many patients. Strategies should be adapted to each local situation, but providing the best imaging test available should remain the priority. Ruling out PE without the use of a ventilation scan is likely safe. However, confirming PE requires a ventilation scan. Otherwise, the referring physician should be advised of the risk of a false-positive result.

## Notes

KEY POINTS **QUESTION:** In COVID-19 patients assessed with lung scintigraphy for suspected acute PE, what is the role of the ventilation scan? **PERTINENT FINDINGS:** In a retrospective review of 145 V/P SPECT/CT scans of COVID-19 patients referred for suspected acute PE, PE could confidently be excluded without the ventilation study in only 57% of patients. In contrast, the ventilation study was required to confidently rule out PE in 31% of patients, confirming the high risk of false-positive results when omitting the ventilation study. **IMPLICATIONS FOR PATIENT CARE:** In COVID-19 patients, confirming a diagnosis of PE with lung scintigraphy requires a ventilation scan.
